# The Glasgow Benefit Inventory—Assessment of Patients Who Underwent Laser-Assisted Dacryocystorhinostomy (LDCR)

**DOI:** 10.3390/jcm13237130

**Published:** 2024-11-25

**Authors:** Radosław Różycki, Katarzyna Ulaszewska, Łukasz Skrzypiec

**Affiliations:** 1 Department of Ophthalmology, Military Institute of Aviation Medicine, 01-755 Warsaw, Poland; radoslawrozycki@gmail.com; 2 “Orbita” Medical Center, 03-808 Warsaw, Poland; 3 Department of Otolaryngology, Military Institute of Medicine—National Research Institute, 04-349 Warsaw, Poland; skrzypieclukas@gmail.com

**Keywords:** lacrimal duct obstruction, LDCR, efficacy, lacrimal surgery, Glasgow Benefit Inventory, patient satisfaction

## Abstract

**Background/Objectives:** Disruptions in lacrimal function can lead to various conditions, including chronic nasolacrimal duct obstruction and dacryocystitis, which may cause symptoms like excessive tearing, pain, and recurrent infections. In cases where pharmacological treatments are insufficient, surgical intervention becomes essential, which is widely used to restore effective tear drainage. **Methods:** This study evaluated postoperative outcomes of laser-assisted dacryocystorhinostomy (LDCR) in 48 patients, totaling 56 treated eyes, over a follow-up period of 6 months to 3.5 years. During the follow-up examination, patients completed the Glasgow Benefit Inventory questionnaire. **Results:** The outcomes demonstrated significant improvements, particularly in the GBI total score (+35.07) and general subscale (+44.36), with minimal effects on social support and physical health subscales. **Conclusions:** LDCR proves to be an effective, minimally invasive alternative to traditional dacryocystorhinostomy, offering significant patient satisfaction, faster recovery, and reduced complications in treating nasolacrimal duct obstruction.

## 1. Introduction

The lacrimal system plays a crucial role in protecting and moisturizing the ocular surface, and its proper function is essential for maintaining eye health and good vision. The lacrimal system consists of the lacrimal glands and the lacrimal drainage system, which includes the lacrimal punctum, lacrimal canaliculus, lacrimal sac, and nasolacrimal duct [1]. These structures work together to produce, transport, and drain tears. Disruptions in the functioning of this system can lead to various conditions, such as dacryocystitis, chronic nasolacrimal duct obstruction, and other disorders that impair tear drainage, resulting in symptoms such as excessive tearing (epiphora), pain, and recurrent infections [2].

The treatment of lacrimal system diseases often requires surgical intervention, especially in cases where pharmacological therapies prove insufficient [3]. The history of surgical treatment dates back to the 18th century in England, where a hole was drilled into the lacrimal bone and a metal tube was inserted to maintain patency. Even then, success rates of 70–85% were achieved [4]. Traditional dacryocystorhinostomy (DCR) is one of the most commonly performed surgeries to restore proper tear drainage by creating a new drainage pathway directly into the nasal cavity. However, this method carries certain risks of complications and a lengthy recovery period. In response to these challenges, medical technology has introduced laser-assisted dacryocystorhinostomy (LDCR), which offers a less invasive alternative [5].

LDCR involves the use of precise laser energy to create a small opening in the nasal bone, establishing a new pathway for tear drainage [6]. This procedure is characterized by its reduced invasiveness, shorter operative time, and faster recovery period compared to traditional surgical methods. Additionally, the laser technique minimizes the risk of complications such as bleeding and infections, making it an increasingly popular choice for treating nasolacrimal duct obstruction [7].

This procedure can be performed on both children and adults, with virtually no age limitations. Currently, according to the available literature, the effectiveness of achieving patency and reducing tearing ranges from approximately 60% [6] to as high as 92.4% [8]. The effectiveness of surgery and patient satisfaction are key indicators in evaluating any medical procedure. For LDCR, analyzing patients’ subjective satisfaction can provide valuable insights into quality of life post-operation, perceived comfort levels, and the overall effectiveness of the procedure.

The aim of this study is to assess the subjective satisfaction of patients following LDCR and its impact on quality of life. The study involved a survey based on the validated Glasgow Benefit Inventory (GBI), with the results intended to summarize patients’ subjective experiences regarding the effectiveness of the surgery, satisfaction levels, and impact on daily functioning. Analyzing these data will enhance the understanding of the benefits and potential limitations of this method, as well as provide essential information for further improving surgical procedures and patient care for those with lacrimal system disorders. This is the second paper in the series [9].

## 2. Materials and Methods

This study presents the results of follow-up examinations conducted on patients who underwent LDCR at the same medical center. Initially, 156 patients meeting the inclusion criteria were selected from the clinic’s database. Of these, 48 patients aged between 29 and 81 years consented to participate in the study and agreed to the use of their data for scientific purposes. A total of 56 eyes underwent the surgical procedure. The surgeries were performed between May 2021 and April 2023, and the postoperative evaluation described in this study was conducted at least 6 months after the surgeries, specifically in September 2023 and November 2023. This provided a postoperative follow-up period ranging from 6 months to 3.5 years, allowing for a more comprehensive assessment of the effectiveness and durability of the LDCR procedure. The surgeries were always performed by the same team, operating together simultaneously. The team included the ophthalmologist Radosław Różycki and otolaryngologist Łukasz Skrzypiec, and surgeries were performed at the “Orbita” Medical Center in Warsaw.

In the surgeries performed, fistulas were created through tissue evaporation at the laser energy application site. For this purpose, a Diomed 15 Plus Diode Surgical Laser Angiodynamics 810 nm ELVT Vascular 2005 (Diomed Inc., Cambridge, UK) was utilized. A protocol was established, based on thermographic studies and the literature, which standardized the average laser power at 10 watts. This standardization aimed to reduce variability and ensure consistency in the results.

During the follow-up visit, (1) formal consent for participation in the study was obtained from the patients and (2) patients were asked to complete a satisfaction survey based on the Glasgow Benefit Inventory. Following these formalities, patients were invited to the office where (3) an interview was conducted and (4) detailed examinations of the lacrimal pathways were performed to assess the effectiveness of the surgical intervention. During the physical examinations, the patency of the lacrimal pathways was assessed using the canalicular test and the reflux test. Additionally, the size and position of the fistula were evaluated, and a fluorescein disappearance test was performed.

According to the Act of 5 December 1996, on the Professions of Doctor and Dentist, ethical approval was not required for this study. The study adheres to the principles of the Helsinki Declaration (1964). Participants did not receive any financial or material benefits for their participation. Information regarding anonymity and the scientific purposes for which the collected data would be used was clearly outlined in the consent forms signed by the patients before the study commenced.

## 3. Results

### 3.1. Patient Characteristics

The study included 33 women (69%) and 15 men (31%), totaling 48 patients. Among the patients, the majority were women; however, the procedure involved a total of 56 eyes—28 right and 28 left. Of these, five eyes had undergone the surgery previously, while the remaining eyes were operated on for the first time. Eight patients had surgery on both the left and right sides simultaneously. The average age of the patients at the time of surgery was 65 years, with ages ranging from 29 to 81 years, reflecting a broad age range of the operated individuals. The median duration of symptoms before the surgery was 3 years, with a range from 6 months to 50 years, and the most common duration was 2 years.

### 3.2. Surgical Success

The LDCR procedure demonstrated a 95% success rate in achieving anatomical success, defined as the presence of a patent fistula in postoperative evaluations. Anatomical success was achieved in fifty-three eyes, while it was not achieved in three eyes (5%). In the group with anatomical success, functional success was also attained, which was defined as a significant reduction in tearing and effective tear drainage. In the group with persistent obstruction, functional success was not observed.

Each patient was assessed using the Munk score both before and after the surgery. The average score before surgery on the Munk score (0–5) was 4.66, indicating that patients experienced symptoms ranging from frequent tear wiping (10 times a day) to continuous tearing, which significantly impacted their quality of life [10]. After surgery, the average score was 0.43, indicating that patients wipe their eyes zero to two times a day.

### 3.3. Survey Satisfaction Results

During the visit, patients were asked to complete a postoperative satisfaction survey using the Glasgow Benefit Inventory (GBI) [11]. This questionnaire is designed to evaluate the impact of the surgical outcome on life and overall well-being. Patients were instructed to select one of five numerical options for each question. The numbers represented the following responses:

1 = Much worse;

2 = A little or somewhat worse;

3 = No change;

4 = A little or somewhat better;

5 = Much better.

Instructions for completing the questionnaire were provided with each form. The results and questions of the survey are presented below in Table 1.

**Figure 1:**
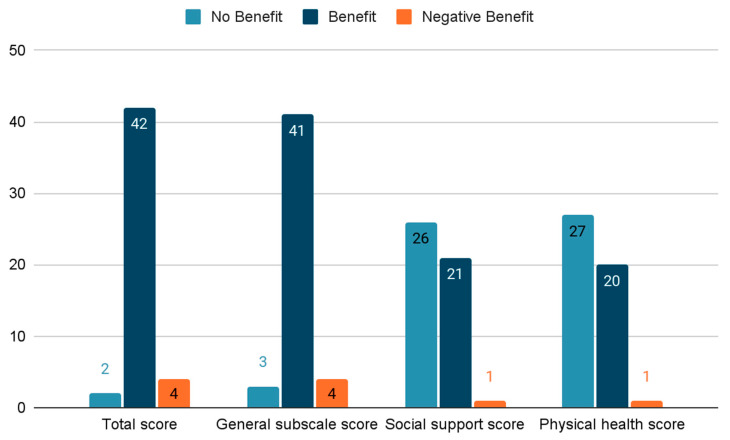
Distribution of patient benefits by category.

The survey results were summarized and categorized into the following scores: total score (questions 1 to 18); general subscale score (questions 1–6, 9, 10, 14, 16–18); social support score (questions 7, 11, 15); and physical health score (questions 8, 12, 13) [12]. The results are presented in Table 2 and Figure 1.

Calculations were performed according to the following formula: TOTAL [(sum of responses/number of questions) − 3] × 50.

The impact of the procedure on various aspects of life can range from −100 (maximum negative impact), through 0 (no impact), to +100 (maximum positive impact).

The time elapsed since the surgery did not reveal a clear correlation between the duration of symptoms and GBI scores or the success rate in our analysis.

## 4. Discussion

The survey was conducted on 48 patients, demonstrating the positive impact of LDCR surgery on patients’ lives, as measured by the GBI. Particularly strong results were observed in the total score (+35.07) and general subscale (+44.36). Most patients did not report significant effects on social support or physical health. Four patients, however, noted negative impacts in certain categories. The social support and physical health subscales may be less sensitive to changes resulting from the procedure, as lacrimal duct obstruction does not significantly impair the activity of patients, nor does it contraindicate interpersonal interactions [13]. On the other hand, the general subscale is more comprehensive and can encompass a wide range of quality of life aspects, which may improve significantly after the procedure, such as daily functionality, emotional well-being, and comfort level.

The GBI, used in this study, is a well-established and validated tool that was first implemented in 1996 [13]. It is designed to assess health benefits from the patient’s perspective, particularly for otorhinolaryngological interventions. The 18-item post-intervention questionnaire is sensitive to various treatments and provides a common metric to compare their outcomes. It enables the profiling of results, which is useful for audits, research, and health policy planning [14]. GBI is considered appropriate for comparing different types of surgeries and can help identify predictors of patient benefits.

LDCR is an advanced surgical technique used to treat obstructions in the lacrimal duct system, which can result in excessive tearing, recurrent infections, and discomfort. By creating a new pathway for tear drainage, LDCR restores normal tear flow and alleviates associated symptoms. Utilizing laser technology, this minimally invasive procedure offers quicker recovery times and less postoperative discomfort compared to traditional methods. Patients often experience significant improvements in quality of life, as the restoration of tear function not only alleviates physical symptoms but also reduces the emotional distress linked to chronic tearing and infections, making LDCR a valuable option for those with lacrimal duct obstructions [9].

In one study, a GBI survey conducted over 6 months post-LDCR on 156 patients produced a mean total score of +16.8 [15]. In our case, this was +32.99.

Additional data suggest varying levels of patient satisfaction depending on the underlying condition. For instance, GBI scores for nasolacrimal duct obstruction were +32.7 (26.3–37.1), for mucocele they were +40.1 (28.7–51.4), and for dacryocystitis they were +19.4 (10.0–28.9) [16].

Yeniad et al. evaluated GBI satisfaction after 1 and 3 months in patients who underwent bilateral external DCR (EXT-DCR) surgery, with scores improving over time: total GBI (+27.8 vs. +37.2), general subscale (+26.2 vs. +35.7), social subscale (+29.1 vs. +41.6), and physical health (+30.4 vs. +49.4) [14]. This highlights the progressive improvement in patients’ well-being. In comparison, after LDCR, our patients achieved even higher satisfaction on the general subscale. The same study conducted an analysis among 19 patients who simultaneously underwent LDCR surgery on one eye and EXT-DCR on the other eye. The quality of life of the patients significantly improved after the surgery, according to the GBI results at 1- and 3-months post-operation. Based on the data we obtained, we can conclude that high satisfaction and surgical success rates were maintained throughout the observation period. However, we are aware that the long-term assessment of the procedure’s effectiveness may be influenced by later complications or symptom recurrence. In our study, no significant recurrences or complications were observed after this period, but we acknowledge that such possibilities cannot be ruled out over a longer duration.

In Southern India, 112 EXT-DCR patients also were evaluated at 1- and 3-months post-surgery, showing improvements across all parameters: total scale (+32.22 vs. +48.86), general subscale (+31.21 vs. +44.08), social support (+46.28 vs. +61.01), and physical health (+22.17 vs. +55.80) [17]. Another study recorded a total score of +42.67 in EXT-DCR patients, with a negative correlation between GBI and Munk score [18].

Among 77 patients, 37 underwent EXT-DCR and 40 underwent endonasal DCR (END-DCR). Both groups showed health improvements, with GBI scores of +16.1 for EXT-DCR and +24.1 for END-DCR, although the difference was not statistically significant [19]. Similarly, END-DCR yielded higher satisfaction (9.3 vs. 8.6) compared to EXT-DCR in another study [20]. However, some research found no significant difference in satisfaction between these procedures [21].

In a study of 54 EXT-DCR patients aged 26–30, GBI results after 6 months showed the following: total score (+48.83), general subscale (+52.70), social support (+49.69), and physical health (+37.07) [22]. A separate 5-year follow-up showed satisfaction decline over time, from 89% after 1 year to 71% after 4–5 years, reflecting the progressive deterioration of surgical outcomes [23].

In 110 patients who underwent endoscopic endonasal DCR (EE-DCR), a retrospective survey conducted at least 6 months post-surgery reported improvements in most aspects, with a total score of +15.04, general subscale score of +22.16, and social support score of +4.67, though physical health scored negatively (−4.47). No differences in outcomes were observed based on the surgeon’s experience, but younger patients achieved better results than older patients [24]. The operations of EE-DCR and EXT-DCR were compared in a telephone survey of patients. The study indicates that patient satisfaction is comparable to the surgical outcomes, with satisfaction rates of 70% to 75% for primary procedures and 63% to 75% for revision procedures using the EE-DCR method. In contrast, the EXT-DCR procedure achieved an 86% satisfaction rate among patients [25].

In our study, a satisfactory outcome was achieved after the LDCR procedure, with a significant reduction of more than 4 points in the Munk scale among patients. When compared to this result, a study on 10 patients who underwent EE-DCR showed an initial Munk score of 1.8, which decreased to 0 postoperatively [26]. Another study involving 60 patients who underwent EE-DCR found that Munk scores were significantly reduced at 6 months and 2 years post-operation compared to preoperative scores, with mean scores of 0.29 ± 0.68, 0.59 ± 1.20, and 3.21 ± 1.32, respectively [27]. Interestingly, better results are observed shortly after the EE-DCR surgery, with both EE-DCR and EXT-DCR achieving similar outcomes in the Munk scale in the following days [28].

These findings indicate that different methods of treating lacrimal duct obstruction can lead to good patient satisfaction, with appropriate approaches tailored to the specific condition.

## 5. Conclusions

The study demonstrated that patients who underwent LDCR surgery reported high levels of satisfaction, as evidenced by the significant positive scores on the GBI. Patients experienced notable improvements in their quality of life following the procedure. The results suggest that LDCR is effective in improving patient-reported outcomes compared to other surgical methods, highlighting its viability as a treatment option for nasolacrimal duct obstruction. Utilizing the GBI as a patient-centered assessment tool provided valuable insights into the subjective benefits experienced by patients, reinforcing its role in evaluating surgical outcomes in ophthalmology. Future studies should aim to explore specific factors influencing patient satisfaction post-LDCR and assess long-term outcomes in a larger cohort for a more comprehensive understanding. These conclusions highlight the effectiveness and patient-centered focus of LDCR surgery, underscoring its role in improving patient satisfaction and overall health outcomes.

In the future, we might consider conducting a more detailed subgroup analysis that takes into account factors such as patient age, severity of obstruction (e.g., mild, moderate, severe), and the duration of symptoms before surgery. This study could involve tracking patient satisfaction over time and correlating it with these variables using appropriate statistical methods, such as multivariate regression analysis. This approach could help identify potential trends or patterns in satisfaction and provide a deeper understanding of the factors influencing postoperative outcomes. Additionally, future studies might benefit from incorporating qualitative feedback from patients, which could offer valuable insights into their individual experiences and further enrich the analysis.

## 6. Limitations

The study has certain limitations that should be considered when interpreting the results. The lack of a control group of patients who did not undergo surgery makes it difficult to compare the obtained results with other treatment methods or the natural course of the disease. Additionally, some patients were not available for postoperative follow-up, which could affect the representativeness of the findings. A larger number of observed patients might provide a more comprehensive view of the situation.

Moreover, differences in follow-up duration and individual postoperative care could have influenced the variability in patient satisfaction outcomes. Therefore, it is suggested to conduct further observations after a longer period to fully assess the durability of the surgical effects and long-term patient satisfaction.

## Figures and Tables

**Table 1:** Results of the satisfaction survey.

Questions	1	2	3	4	5
Has the result of the operation affected the things you do?	0 (0%)	5 (10.42%)	6 (12.5%)	12 (25%)	25 (52.08%)
Have the results of the operation made your overall life better or worse?	1 (2.08%)	1 (2.08%)	5 (10.42%)	10 (20.83%)	31 (64.58%)
Since the operation, have you felt more or less optimistic about the future?	1 (2.08%)	2 (4.17%)	11 (22.92%)	13 (27.08%)	21 (43.75%)
Since your operation, do you feel more or less embarrassed when with a group of people?	1 (2.08%)	1 (2.08%)	18 (37.5%)	5 (10.42%)	23 (47.23%)
Since your operation, do you have more or less self-confidence?	0 (0%)	1 (2.08%)	21 (43.75%)	8 (16.67%)	18 (37.5%)
Since your operation, have you found it easier or harder to deal with the company?	0 (0%)	1 (2.08%)	23 (47.23%)	7 (14.58)	17
Since your operation, do you feel that you have more or less support from your friends?	0 (0%)	0 (0%)	35 (72.92%)	6 (12.5%)	7 (14.58)
Have you been to your family doctor, for any reason, more or less often, since your operation?	1 (2.08%)	1 (2.08%)	36 (75%)	4 (8.33%)	6 (12.5%)
Since your operation, do you feel more or less confident about job opportunities?	0 (0%)	1 (2.08%)	27 (56.25%)	7 (14.58)	13 (27.08%)
Since your operation, do you feel more or less self-conscious?	1 (2.08%)	1 (2.08%)	20 (41.67%)	7 (14.58)	19 (39.58%)
Since your operation, are the more or fewer people who really care about you?	0 (0%)	0 (0%)	35 (72.92%)	5 (10,42%)	8 (16.67%)
Since you had the operation, do you catch colds or infections more or less often?	2 (4.17%)	1 (2.08%)	32 (66.67%)	4 (8.33%)	9 (18.75%)
Have you had to take more or less medicine for any reason, since your operation?	1 (2.08%)	0 (0%)	39 (81.25%)	4 (8.33%)	4 (8.33%)
Since your operation, do you feel better or worse about yourself?	0 (0%)	3 (6.25%)	11 (22.92%)	9 (18.75%)	25 (52.08%)
Since your operation, do you feel that you have had more or less support from your family?	0 (0%)	1 (2.08%)	38 (79.17%)	3 (6.25%)	6 (12.5%)
Since your operation, are you more or less inconvenienced by your health problem?	3 (6.25%)	3 (6.25%)	15 (31.25%)	6 (12.5%)	21 (43.75%)
Since your operation, have you been able to participate in more or fewer social activities?	1 (2.08%)	0 (0%)	32 (66.67%)	6 (12.5%)	9 (18.75%)
Since your operation, have you been more or less inclined to withdraw from social situations?	1 (2.08%)	2 (0%)	35 (72.92%)	4 (8.33%)	6 (12.5%)

**Table 2:** Results of satisfaction survey calculations.

Category	Mean Score	Highest Score	Lowest Score
Total Score	+35.07	+91.67	−22.22
General subscale score	+44.36	+100	−41.67
Social support score	+19.10	+100	−16.67
Physical health score	+13.88	+100	−100

## Data Availability

The data collected in the study are available at the Orbita Medical Center, reachable via email at kontakt@centrummedyczneorbita.pl.
